# Sustained virological response after treatment with direct antiviral agents in individuals with HIV and hepatitis C co‐infection

**DOI:** 10.1002/jia2.26048

**Published:** 2022-12-23

**Authors:** Sara Lodi, Marina Klein, Andri Rauch, Rachel Epstein, Linda Wittkop, Roger Logan, Christopher T. Rentsch, Amy C. Justice, Giota Touloumi, Juan Berenguer, Inma Jarrin, Matthias Egger, Massimo Puoti, Antonella D'Arminio Monforte, John Gill, Dominique Salmon Ceron, Ard van Sighem, Benjamin Linas, Marc van der Valk, Miguel A. Hernán, HepCAUSAL Collaboration

**Affiliations:** ^1^ Department of Biostatistics Boston University School of Public Health Boston Massachusetts USA; ^2^ CAUSALab, Harvard T.H. Chan School of Public Health Boston Massachusetts USA; ^3^ Division of Infectious Diseases and Chronic Viral Illness Service Department of Medicine McGill University Montreal Quebec Canada; ^4^ Department of Epidemiology Biostatistics and Occupational Health McGill University Montreal Quebec Canada; ^5^ Department of Infectious Diseases Inselspital Bern University Hospital University of Bern Bern Switzerland; ^6^ Department of Pediatrics Section of Infectious Diseases Boston University School of Medicine Boston Massachusetts USA; ^7^ Department of Medicine Section of Infectious Diseases Boston University School of Medicine Boston Massachusetts USA; ^8^ ISPED, INSERM Bordeaux Population Health Research Center University of Bordeaux Bordeaux France; ^9^ CHU de Bordeaux Pôle de Santé Publique Bordeaux France; ^10^ Department of Epidemiology Harvard T.H. Chan School of Public Health Boston Massachusetts USA; ^11^ Department of Internal Medicine Yale School of Medicine New Haven Connecticut USA; ^12^ VA Connecticut Healthcare System US Department of Veterans Affairs New Haven Connecticut USA; ^13^ Faculty of Epidemiology and Population Health London School of Hygiene & Tropical Medicine London UK; ^14^ Department of Health Policy Yale School of Public Health New Haven Connecticut USA; ^15^ Department of Hygiene Epidemiology & Medical Statistics Medical School National & Kapodistrian University of Athens Athens Greece; ^16^ Hospital General Universitario Gregorio Marañón Madrid Spain; ^17^ Centro Nacional de Epidemiologia Institute of Health Carlos III Madrid Spain; ^18^ Institute of Social and Preventive Medicine University of Bern Bern Switzerland; ^19^ School of Medicine and Surgery University of Milan Bicocca – ASST GOM Niguarda Milan Milano Italy; ^20^ Clinic of Infectious Diseases Department of Health Sciences ASST Santi Paolo e Carlo Milan Italy; ^21^ Southern Alberta Clinic Calgary Alberta Canada; ^22^ Department of Medicine University of Calgary Calgary Alberta Canada; ^23^ Department of Infectious Diseases and Immunology Hotel Dieu Hospital Paris Public Hospitals (APHP) Paris France; ^24^ School of Medicine University of Paris Paris France; ^25^ Stichting HIV Monitoring Amsterdam The Netherlands; ^26^ Boston Medical Center and Epidemiology Boston Massachusetts USA; ^27^ Boston University Schools of Medicine and Epidemiology Boston Massachusetts USA; ^28^ Department of Internal Medicine Amsterdam Infection and Immunity Institute and Amsterdam Public Health Research Institute Amsterdam The Netherlands; ^29^ University of Amsterdam Amsterdam The Netherlands; ^30^ Department of Biostatistics Harvard T.H. Chan School of Public Health Boston Massachusetts USA

**Keywords:** HIV and HCV co‐infection, direct antiviral agents, sustained virological response, causal inference, parametric g‐formula, missing data

## Abstract

**Introduction:**

Randomized trials and observational studies have consistently reported rates of sustained virological response (SVR), equivalent to hepatitis C virus (HCV) cure, as high as 95% following treatment with direct‐acting antiviral (DAA) treatment in individuals with HIV and HCV co‐infection. However, large studies assessing whether SVR rates differ according to demographic and clinical strata are lacking. Additionally, the SVR rates reported in the literature were typically computed in non‐random samples of individuals with available post‐DAA HCV‐RNA measures. Here, we aimed to estimate the probability of SVR after DAA treatment initiation in persons with HIV and HCV co‐infection overall and by demographic and clinical characteristics with and without adjustment for missing HCV‐RNA testing.

**Methods:**

We included adults with HIV‐HCV co‐infection who received DAA treatment between 2014 and 2020 in HepCAUSAL, an international collaboration of cohorts from Europe and North America. We estimated the proportions of DAA recipients who had documented SVR (defined as an undetectable HCV‐RNA at least 12 weeks after the end of DAA treatment) overall and by strata defined by age, sex, presence of cirrhosis, calendar period, mode of HIV acquisition, CD4 cell count and HCV genotype at DAA treatment. We then compared these rates with those obtained using the parametric g‐formula to impute SVR status for individuals with no SVR assessment.

**Results and Discussion:**

A total of 4527 individuals who initiated DAA treatment (88% males, median [IQR] age 56 [50, 62] years) were included. Of the total of 642 (14%) individuals had no HCV‐RNA test on or after 12 weeks after the end of treatment. The overall observed and g‐formula imputed SVR rates were 93% (95% CI 93, 94) and 94% (95% CI 92, 95), respectively. SVR estimates were similarly high across all strata. A substantial proportion of individuals who received DAA treatment were never assessed for SVR post‐DAA and strategies for more systematic routine HCV‐RNA testing should be considered.

**Conclusions:**

Our estimates with and without adjustment for missing HCV‐RNA testing indicate SVR rates of approximately 95%, like those reported in clinical trials.

## 1 INTRODUCTION

Direct‐acting antiviral (DAA) treatment has revolutionized the treatment of hepatitis C virus (HCV) infection. The availability of DAAs is especially important for persons with HIV because HCV co‐infection, which is common among injection drug users and men who have sex with men [1, 2, 3], can lead to accelerated progression of liver disease, a major cause of death in these groups [4, 5].

Over 95% of individuals with HIV and HCV co‐infection treated with DAA treatment achieved sustained virological response (SVR), defined as the absence of HCV viremia, in both randomized trials [6, 7, 8, 9, 10, 11, 12, 13] and observational studies [14, 15, 16, 17, 18, 19, 20, 21]. However, existing studies lack a sufficient sample size to provide precise subgroup estimates to characterize differences in SVR rates by age, presence of cirrhosis, HCV genotype and immunosuppression, which were risk factors for HCV treatment failure in the pre‐DAA era. Also, most of these studies included patients treated during the first wave of DAAs available between 2014 and 2016, who were possibly a selected group with more advanced liver fibrosis or more likely to engage in treatment. New studies with recent follow‐up are, therefore, needed to examine whether SVR proportions have remained similarly high in recent years after DAA treatment became widely available to individuals without HCV‐related comorbidities or newly diagnosed with HCV.

Determining SVR, that is the absence of HCV viremia 12 weeks after the end of treatment, requires that an HCV‐RNA testing is performed at 12 weeks after treatment ends. Therefore, observational analyses are typically restricted to individuals with at least one HCV‐RNA test 12 or more weeks after treatment. This approach, however, may result in biased estimates of SVR rates if tested individuals are not a random sample of all treated individuals and it also does not allow any differentiation between treatment failure and HCV re‐infection in those individuals whose first test occurs months after the end of treatment and is positive.

Here, we aimed to: (1) estimate the probability of SVR after DAA treatment initiation between 2014 and 2020, overall and by subgroups defined by sex, age, cirrhosis, mode of HIV acquisition, CD4 cell count and calendar year of DAA initiation using a large international consortium of cohorts of individuals with HIV and HCV co‐infection and (2) compare these proportions with those obtained using the parametric g‐formula to impute SVR status for individuals with no SVR assessment.

## 2 METHODS

### 2.1 Study data

The HepCAUSAL Collaboration is a consortium of prospective cohorts of individuals with HIV and HCV co‐infection from six European countries, the United States and Canada. All cohorts record routinely collected data in clinical practice in settings with universal access to care. The data include demographics, region of birth, vitals, mode of HIV transmission, laboratory measurements, history of HCV and HIV treatment, diagnosis of AIDS and non‐AIDS‐defining events, date of death and hepatitis B co‐infection.

Data are submitted in a standardized format (http://www.hicdep.org/) by each cohort and are harmonized at the coordinating centre. The analyses presented here are based on data pooled from the following nine cohorts: Aquitaine (France), AMACS (Greece), ICONA (Italy), ATHENA (Netherlands), CoRIS (Spain), Swiss HIV Cohort Study (Switzerland), HIV Boston Medical Center cohort (US), Veterans Aging Cohort Study (VACS) (US) and the Canadian Co‐infection Cohort study (Canada).

Research based on these data was approved by the Institutional Review Board (IRB) of the Harvard TH Chan School of Public Health; the last review took place on August 2020. All participating cohorts received approval from their local IRB.

### 2.2 Eligibility criteria

Individuals were eligible if they initiated their first course of DAA (a regimen containing sofosbuvir, ledipasvir, daclatasvir, simeprevir, velpatasvir, glecaprevir, pibrentasvir, grazoprevir, ombitasvir, paritaprevir or voxilaprevir) with or without ribavirin between 2014 and 2020 and met the following criteria: age≥18 years; alanine transaminase (ALT), aspartate transaminase (AST) and platelet count (the laboratory components of the Fibrosis‐4 [FIB‐4] score [22]) measured less than 6 months before DAA initiation, CD4 cell count and HIV‐RNA measured less than 12 months before DAA initiation. Because most patients received sofosbuvir/ledipasvir or other combinations that typically require 12 weeks duration, for patients with unknown end‐of‐treatment date, we assumed a treatment duration of 12 weeks.

### 2.3 Outcome

SVR was defined as a negative HCV‐RNA test (viral load below the level of detection or <25 IU/ml when the lower limit of detection was unknown) at or after 12 weeks after the end of treatment. Re‐initiation of DAA before 23 weeks after the end of treatment was considered a failure to achieve SVR.

### 2.4 Statistical analysis

We calculated the proportions of individuals who achieved SVR using two methods.

First, we restricted the calculations to individuals with an HCV‐RNA test on or after 12 weeks after the end of treatment. We estimated the proportion of SVR overall and in subgroups defined by sex (female and male), age (≤50 and >50 years), FIB‐4 at baseline (≤3.25 vs. >3.25), AST to platelet ratio index (APRI) at baseline (<1.5 vs. ≥1.5), mode of HIV acquisition (sexual contact, injection drug use and unknown), CD4 cell count category (<350, 350–500 and ≥500 cells/mm^3^), HCV genotype (1, 2, 3 or other) and calendar period of DAA initiation (2014–2016 and 2017–2020). We also reported SVR rates by HCV genotype and cirrhosis strata.

Second, we repeated the calculations with the imputation of SVR status between 12 and 23 weeks among 14% of individuals who had no HCV‐RNA test on or after 12 weeks after the end of treatment overall and by all subgroups except for HCV genotype due to the low number of individuals with a non‐1 genotype.

To impute SVR status, we used the parametric g‐formula with adjustment for the following variables measured at DAA initiation: age (<35, 35–50 and >50 years), sex, mode of HIV acquisition, cohort, calendar period, CD4 cell count category (<350, 350–500 and >500 cells/mm^3^), HIV‐RNA (≤50 vs. >50 copies/ml), history of antiretroviral treatment for HIV, history of AIDS, hepatitis B virus co‐infection (presence of either hepatitis B surface antigen or detectable hepatitis B virus DNA), prior HCV treatment with interferon, HCV genotype and fibrosis stage categorized as no significant fibrosis (FIB‐4<1.45), significant fibrosis (FIB‐4≥1.45 and FIB‐4≤3.25) and cirrhosis (FIB‐4>3.25) [23, 24].

Because individuals who engaged more in HCV care may be more likely to adhere to DAA and to be assessed for SVR, we also adjusted for the following prognostic factors that are markers for engagement in care: log‐transformed time‐varying AST, ALT and platelet count values (components of the FIB‐4 score), time‐varying indicators for AST, ALT and platelet count testing, and HCV‐RNA testing during DAA treatment (yes, no), as some guidelines recommended testing at 4 weeks after DAA treatment initiation [25] for most of the study period.

The parametric g‐formula estimation procedure has been described elsewhere [26, 27, 28]. Briefly, we followed two steps (Supplementary Material). First, we fit parametric regression models to estimate the joint distribution of the outcome (SVR status), time‐varying HCV‐RNA testing and time‐varying covariates in the SVR assessment period conditional on the baseline variables and on the history of the time‐varying covariates in the SVR assessment window. Second, we ran a Monte Carlo simulation using the above estimates to simulate the distribution of the SVR rates under an intervention that imposes at least one HCV‐RNA test during the SVR assessment period. We used a non‐parametric bootstrap procedure based on 500 samples to obtain percentile‐based 95% confidence intervals. To ascertain the validity of our parametric assumptions, we checked that the observed means of the post‐baseline covariates were similar to those predicted by our models (not shown). All analyses were conducted with the publicly available SAS macro GFORMULA [29].

## 3 RESULTS AND DISCUSSION

The study included 4527 eligible individuals, of whom 88% were males, 85% received DAA treatment between 2014 and 2016, 95% were ART‐experienced and 89% had undetectable HIV‐RNA (Table 1 and Table S1). Median [IQR] age, CD4 count and body mass index (BMI) at DAA initiation were 56 [50, 62] years, 570 [387, 789] cells/mm^3^ and 21 [19, 24], respectively. Consistent with clinical practice in Europe and North America during the study time, most patients (57%) received ledipasvir‐sofosbuvir. Among 3339 individuals with an available HCV genotype, 2767 (83%) had genotype 1. Compared with individuals who received DAA treatment in 2014–2016, those who received DAA in 2017–2020 were younger, more likely to have a non‐1 HCV genotype, a lower FIB‐4 score and injection drug use as a mode of HIV transmission (Table 2).

**Table 1:** Baseline characteristics of 4527 patients with hepatitis C and HIV co‐infection who initiated direct‐acting antiviral (DAA) treatment

Characteristics at DAA initiation	Eligible patients (%)	Assessed for SVR on or after 12 weeks after the end of treatment (% of eligible patients)
*Sex*		
Female	531 (12%)	434 (82%)
Male	3996 (88%)	3451 (86%)
*Age, years*		
≤50	1182 (26%)	999 (85%)
>50	3345 (74%)	2886 (86%)
*FIB‐4 score*		
≤3.25	3451 (76%)	2969 (86%)
>3.25	1076 (24%)	916 (85%)
*APRI*		
≤1.5	3702 (82%)	3191 (82%)
>1.5	825 (18%)	694 (84%)
*Year of DAA initiation*		
2014–2016	3843 (85%)	3351 (87%)
2017–2020	684 (15%)	534 (78%)
*Mode of HIV acquisition*		
Heterosexual contact	250 (5%)	195 (78%)
Homosexual contact	502 (11%)	410 (82%)
Injection drug use	991 (22%)	801 (81%)
Other/unknown	2784 (62%)	2479 (89%)
*Region of birth*		
High‐income country	3912 (86%)	3431 (88%)
Low‐ or mid‐income country	145 (3%)	127 (86%)
Unknown country	470 (10%)	327 (70%)
*CD4 cell count, cells/mm^3^*		
<350	925 (20%)	788 (85%)
350–500	865 (19%)	731 (85%)
>500	2737 (61%)	2366 (86%)
*HIV‐RNA, copies/ml*		
≤50	4036 (89%)	3461 (86%)
>50	491 (11%)	424 (86%)
*At least one HCV‐RNA test during DAA treatment*		
No	566 (12%)	323 (57%)
Yes	3961 (88%)	3562 (90%)
*Prior interferon treatment*		
No	3551 (78%)	3041 (86%)
Yes	976 (22%)	844 (87%)
*Prior ART initiation*		
No	235 (5%)	205 (87%)
Yes	4292 (95%)	3680 (86%)
*Body mass index*		
≤30 kg/m^2^	4071 (90%)	3504 (86%)
>30 kg/m^2^	199 (4%)	171 (86%)
Unknown	257 (6%)	210 (82%)
*Hepatitis B infection*		
No	4056 (90%)	3477 (86%)
Yes	113 (2%)	98 (87%)
Unknown	358 (8%)	310 (87%)
*Hepatitis C genotype*		
1 (a,b or c)	2767 (61%)	2474 (89%)
2	162 (4%)	142 (88%)
3	273 (6%)	238 (87%)
4	136 (3%)	112 (82%)
Other/unknown	1189 (26%)	919 (77%)
*DAA treatment*		
Ledipasvir‐sofosbuvir	2574 (57%)	2272 (88%)
Sofosbuvir	541 (12%)	411 (76%)
Velpatasvir‐sofosbuvir	342 (8%)	286 (84%)
Daclatasvir+sofosbuvir	272 (6%)	238 (88%)
Ombitasvir‐paritaprevir‐ritonavir+dasabuvir	204 (5%)	169 (83%)
Simeprevir+sofosbuvir	153 (3%)	127 (83%)
Elbasvir‐grezoprevir	150 (3%)	134 (89%)
Glecaprevir‐pibrentasvir	81 (2%)	68 (84%)
Daclatasvir	75 (2%)	63 (84%)
Boceprevir	44 (<1%)	42 (95%)
Ombitasvir‐paritaprevir‐ritonavir	37 (<1%)	30 (81%)
Simeprevir	21 (<1%)	19 (90%)
Telaprevir	21 (<1%)	19 (90%)
Dasabuvir	11 (<1%)	7 (64%)
Sofosbuvir‐velpatasvir‐voxilaprevir	1 (<1%)	0 (0%)
*All*	4527 (100%)	3885 (86%)

Note: HepCAUSAL 2014–2020.

**Table 2:** Baseline characteristics of 4527 patients with hepatitis C and HIV co‐infection who initiated direct‐acting antiviral (DAA) treatment by calendar period of DAA treatment initiation

	Year of DAA initiation	
Characteristics at DAA initiation	2014–2016 *N* (%)	2017–2020 *N* (%)
*All patients*	3843 (100%)	684 (100%)
*Sex*		
Female	352 (9%)	179 (26%)
Male	3491 (91%)	505 (74%)
*Age, years*		
≤50	901 (23%)	281 (41%)
>50	2942 (77%)	403 (59%)
*Mode of HIV acquisition*		
Heterosexual contact	176 (5%)	74 (11%)
Homosexual contact	390 (10%)	112 (16%)
Injection drug use	678 (18%)	313 (46%)
Other/unknown	2599 (68%)	185 (27%)
*CD4 cell count, cells/mm^3^*		
<350	800 (21%)	125 (18%)
350–500	756 (20%)	109 (16%)
>500	2287 (60%)	450 (66%)
HIV‐RNA, copies/ml		
≤50	3421 (89%)	615 (90%)
>50	422 (11%)	69 (10%)
*At least one HCV‐RNA test during DAA treatment*		
No	413 (11%)	153 (22%)
Yes	3430 (89%)	541 (78%)
*Prior interferon treatment*		
No	2947 (78%)	603 (88%)
Yes	896 (23%)	80 (12%)
*Prior ART initiation*		
No	173 (4%)	62 (9%)
Yes	3670 (96%)	622 (91%)
*Prior AIDS diagnosis*		
No	3191 (83%)	560 (82%)
Yes	652 (17%)	124 (18%)
*Hepatitis C genotype*		
1(a,b or c)	2476 (64%)	291 (43%)
2	137 (4%)	25 (4%)
3	170 (4%)	103 (15%)
4	86 (2%)	50 (8%)
Other/unknown	974 (25%)	215 (31%)
*FIB‐4 score*		
≤3.25	2846 (74%)	605 (88%)
>3.25	997 (26%)	79 (12%)

Note: HepCAUSAL 2014–2020.

Of the 4527 eligible individuals, 55 (1%) died, 30 (<1%) received a new DAA prescription by 23 weeks after the end of treatment and 3885 (86%) had at least one HCV‐RNA test at or later than 12 weeks after the end of treatment. SVR testing occurred at a median time [IQR] of 15 [13, 23] weeks after the end of treatment. Only 185 individuals (5%) were tested 1 year after the end of treatment. Of these late testers, 30 (16%) had a detectable HCV RNA test.

The estimated SVR proportions were 93% (95% confidence intervals [CI] 93, 94) among individuals who had at least one HCV‐RNA test and 94% (92, 95) after the imputation of a missing test via the g‐formula (Table 3). The SVR proportion estimates across groups defined by age group, sex, FIB‐4 strata, APRI strata, calendar period, mode of HIV acquisition and CD4 cell count were higher than 92% and were similar under the two methods. SVR rates were also high (95% [65, 96]) among 66 individuals with FIB‐4>3.25 and genotype 3, considered the most challenging HCV genotype to treat.

**Table 3:** Observed and g‐formula estimated sustained virological response (SVR) rates by age, cirrhosis status defined by fibrosis‐4 (FIB‐4) and AST to platelet ratio index (APRI), calendar year and CD4 cell count at initiation of direct‐acting antiviral (DAA) treatment

	SVR rates (95% CI)	
	Standard^a^	Estimated
Overall (*N* = 4527)	93% (93, 94)	94% (92, 95)
*Age (years)*		
≤50 (*N* = 1182)	93% (92, 95)	94% (91, 96)
>50 (*N* = 3345)	93% (93, 94)	93% (91, 95)
*Sex*		
Female (*N* = 531)	93% (92, 94)	94% (84, 96)
Male (*N* = 3996)	94% (92, 96)	94% (92, 95)
*FIB‐4*		
≤3.25 (*N* = 3451)	94% (93, 95)	94% (92, 95)
>3.25—cirrhosis (*N* = 1076)	92% (90, 94)	92% (88, 95)
*APRI*		
≤1.5 (*N* = 3702)	94% (93, 95)	94% (92, 95)
>1.5 (*N* = 825)	92% (89, 94)	92% (87, 95)
*Calendar period*		
2015–2016 (*N* = 3843)	93% (92, 94)	93% (91, 95)
2016–2020 (*N* = 684)	94% (92, 96)	96% (93, 97)
*Mode of HIV acquisition* ^b^		
Sexual contact (*N* = 752)	94% (90, 97)	97% (93, 98)
Injection drug use (*N* = 991)	92% (90, 94)	93% (87, 95)
Other/unknown (*N* = 442)	92% (89, 95)	94% (87, 97)
*CD4 cell count, cell/mm^3^*		
≤350 (*N* = 925)	93% (91, 94)	94% (88, 96)
351–500 (*N* = 865)	94% (92, 95)	94% (89, 96)
>500 (*N* = 2737)	94% (93, 95)	94% (92, 96)

Note: G‐formula estimates imposing a test within 12 and 23 weeks after the end of treatment. HepCAUSAL 2014–2020.

^a^ Observed sustained virological response (SVR) rates were computed as the proportion of individuals with an undetectable HCV‐RNA viral load ≥12 weeks after the end of treatment. 95% confidence intervals are estimated based on a binomial distribution of SVR.

^b^ Homosexual contact and heterosexual contact were grouped together as sexual contact. We excluded the Veterans Ageing Cohort Study because mode of HIV acquisition was unknown in all participants.

Overall, our SVR rates are similar in magnitude to those reported in clinical trials and other observational studies [8, 9, 14, 15, 16, 17, 19, 30, 31, 32]. In particular, we estimated a 95% SVR rate in individuals who received treatment in more recent years (2017–2020), despite a higher prevalence of history of injection drug use, a factor associated with DAA treatment failure [33]. This is reassuring and supports current guidelines recommending universal DAA initiation regardless of substance use.

Because 14% of individuals who received DAA treatment were never assessed for SVR and 5% were assessed after 1 year after the end of treatment, we also estimated SVR rates imputing missing data on SVR status. This method relies on weaker conditions than the analysis restricted to individuals with an HCV‐RNA test [34]. We found that the SVR rates were very similar to those computed restricting the analysis to individuals who had an HCV‐RNA test on or after 12 weeks after the end of treatment. The reasons for the results’ similarity might be due to SVR rates being consistently high in patients with different baseline characteristics. Alternatively, we might have missed important common causes between SVR testing and SVR achievement.

Our study has some limitations. First, the FIB‐4 and APRI are imperfect measurements of the liver fibrosis stage, and they might fail to correctly distinguish between no fibrosis and mild fibrosis. Liver elasticity measurement, a better method to estimate liver fibrosis, was not collected in one of the cohorts and was missing on one‐fourth of the patients in the remaining cohorts. Second, only 10% of the patients had started DAA in recent years and we could not look at subgroups analyses by age, sex, CD4 count and cirrhosis. Third, a small number of individuals (1%) received boceprevir or telaprevir, DAA treatments with relatively low SVR rates, but this is unlikely to have impacted our estimates [35]. Fourth, it has been previously showed that persons who inject drugs are under‐represented in HIV cohorts [36]. Therefore, our SVR estimates may not be generalized to all populations with HIV‐HCV co‐infection in Europe and North America. Fifth, a substantial proportion had an unknown mode of HIV acquisition. Finally, the reasons for the virologic failure of DAA treatment are heterogeneous and include non‐adherence and insufficient drug concentrations due to intestinal malassimilation or impaired cellular uptake [25, 37]. Though we did not have any information on these potential factors, our results indicate that SVR rates are high irrespective of host or viral characteristics.

## 4 CONCLUSIONS

In this study of individuals with HIV and HCV co‐infection who received DAA treatment between 2014 and 2020 in Europe, the United States and Canada as part of their routine clinical care, we estimated that 94% achieved SVR. The SVR rates were similar across subgroups defined by age, CD4 cell count, cirrhosis, HCV genotype and HIV transmission group.

## COMPETING INTERESTS

MK reports grants from ViiV Healthcare, Abbvie and Gilead outside this work; and honoraria for advisory boards from ViiV Healthcare and Abbvie. JG has received honoraria for participation as an ad hoc member of Canadian National HIV Advisory Boards to Merck Gilead and ViiV. GT has received grants unrelated to this study and paid to her institution from Gilead Sciences Europe, UCL, ECDC, EU and National funds. MAH reports consultancy fees from Cytel and ProPublica. No other conflict to report.

## AUTHORS’ CONTRIBUTIONS

Conception: SL; Study design: SL and MH; Acquisition of data: SL, MK, AR, RE, LW, RL, CTR, ACJ, GT, JB, IJ, ME, MP, AD'AM, JG, DSC, AS, BL and MV; Statistical analysis: SL and RL; Interpretation of the data: all authors; Drafted the article: SL; Reviewing of the article: all authors; Critical revision for important intellectual content: all authors; Final approval of the submitted version: all authors.

## FUNDING

This research was supported by the National Institutes of Health grant R37 AI102634 and by the Providence/Boston Center for AIDS Research (P30AI042853).

## Supporting information


**Supplementary Material**: Members of the HepCAUSAL Collaboration.

## Data Availability

Restrictions apply to the availability of these data, due to data user agreements between Harvard and each institution contributing data to HepCAUSAL. The data that support the findings of this study can be requested to each participating cohort.
