# Supplementary material for: Sustained virological response after treatment with direct antiviral agents in individuals with HIV and hepatitis C co‐infection
Source: J Int AIDS Soc. 2022 Dec 23;25(12):e26048. doi: 10.1002/jia2.26048 (PMC9784654; doi:10.1002/jia2.26048)
Supplement: Supplementary file 1 — Supplementary Material: Members of the HepCAUSAL Collaboration. [file JIA2-25-e26048-s001.pdf]

Supplementary online material

Contents

Author information 1
Parametric g-formula 7
Appendix table 1 9

Author information

The HepCAUSAL Collaboration

AQUITAINE
Principal investigator: Pr F. Dabis. Scientific committee: Prs F. Bonnet, D. Breilh, F. Dabis, M. Dupon, G. Chêne, H. Fleury, D. Malvy, P. Mercié, I. Pellegrin, P. Morlat, D. Neau, JL. Pellegrin, R. Thiébaut; Drs S. Bouchet, V. Gaborieau, D. Lacoste, S. Tchamgoué. Epidemiology and biostatistics: Prs G. Chêne, F. Dabis, R. Thiébaut, Drs M. Bruyand, S. Lawson-Ayayi, L. Wittkop. Clinical and biological hospital units: Bordeaux University Hospital: Pr P. Morlat (Pr F. Bonnet, Drs N. Bernard, M. Hessamfar, D. Lacoste, MA. Vandenhende); Pr M. Dupon (Drs FA. Dauchy, H. Dutronc), Pr M. Longy-Boursier (Pr P. Mercié, Drs P. Duffau, J. Roger Schmeltz), Pr D. Malvy (Drs T. Pistone, MC Receveur), Pr D. Neau (Drs C. Cazanave, A. Ochoa, MO. Vareil), Pr JL. Pellegrin (Pr JF. Viallard, Drs C. Greib, E. Lazaro); Pr H. Fleury (Pr ME. Lafon, Drs S. Reigadas, P. Trimoulet); Pr D. Breilh; Pr M. Molimard (Drs S. Bouchet, K. Titier); Pr JF. Moreau (Dr I. Pellegrin); Drs F. Haramburu, G. Miremont-Salamé. Arcachon Hospital: Dr A. Dupont. Dax Hospital: Dr Y. Gerard (Drs L. Caunègre, K. André). Bayonne Hospital: Dr F. Bonnal (Drs S. Farbos, MC. Gemain). Libourne Hospital: Dr J. Ceccaldi (Dr S. Tchamgoué). Mont-de-Marsan Hospital: Dr S. De Witte (Dr C. Courtault). Pau Hospital: Drs E. Monlun (Dr V. Gaborieau). Périgueux Hospital: Dr P. Lataste (Dr JP. Meraud). Villeneuve-sur-Lot Hospital: Dr I. Chossat. Permanent team: MJ. Blaizeau, M. Bruyand, V. Conte, M. Decoin, J. Delaune, S. Delveaux, F. Diarra, C. D'Ivernois, A. Frosch, S. Geffard, C. Hannapier, S. Lawson-Ayayi, E. Lenaud, O. Leleux, F. Le Marec, J. Leray, I. Louis, G. Palmer, A. Pougetoux, X. Sicard, D. Touchard B. Uwamaliya-Nziyumvira.

AMACS
Steering Committee: Adamis G., Antoniadou A., Chini M., Chrysos G., Gikas A., Gogos H.A., Katsarou O., Lazanas M., Metallidis S., Panagopoulos P., Paparizos V., Papastamopoulos V., Paraskevis D., Psychogiou M., Sambatakou H. (Co-Chair), Sipsas NV., Touloumi G. (Chair).
Coordinating Center:
Department of Hygiene, Epidemiology and Medical Statistics, Medical School, National and Kapodistrian University of Athens, Greece (Touloumi G., Pantazis N., Vourli G., Thomadakis C.)
Participating Centers: 4th Dept of Internal Medicine, Medical School, National and Kapodistrian University of Athens, Attikon University Hospital (Antoniadou A, Papadopoulos A); Infectious Disease Unit, "Tzaneio" General Hospital of Piraeus (Chrysos G, Nitsotolis T); 1st Dept of Propedeutic Medicine, Athens University, Medical School "Laikon" General Hospital (Psychogiou M); 1st Dept of Medicine, Infectious Diseases Unit, "G. Gennimatas" Athens General Hospital (Adamis G, Astriti M); 1st Dept of Internal Medicine, Infectious Diseases Section, Patras University Hospital (Gogos HA, Marangos MN);

Blood Transfusion Unit, and National Reference Centre for Congenital Bleeding Disorders, Laikon General Hospital (Katsarou O, Kouramba A); Infectious Diseases Unit, Department of Pathophysiology, General Hospital of Athens “Laikon” and Medical School, National and Kapodistrian University of Athens, Athens, Greece (Sipsas NV, Kontos A); Infectious Diseases Unit, Red Cross General Hospital of Athens (Chini M, Lioni A); First Internal Medicine Department, Infectious Diseases Division, Medical School, Aristotle University of Thessaloniki (Metallidis S, Tsachouridou O); AIDS Unit, Clinic of Venereologic & Dermatologic Diseases, Athens University, Medical School, Syngros Hospital (Paparizos V, Kourkounti S); HIV Unit, 2nd Dept. of Internal Medicine, Athens University, Medical School, Hippokration General Hospital (Sambatakou H); Infectious Diseases & HIV Division, Dept of Internal Medicine, Evaggelismos Athens General Hospital (Papastamopoulos V); Infectious Diseases Unit, University General Hospital of Alexandroupolis, Democritus University of Thrace (Panagopoulos P, Ganitis A); Department of Internal Medicine, University Hospital of Heraklion, Heraklion, Crete, Greece (Gikas A, Barbounakis E).
Hellenic Society for the Study and Control of AIDS: Lazanas M. (Chair), Gogos H. (Co-Chair)

<u>ATHENA</u>
The ATHENA cohort is managed by Stichting HIV Monitoring and supported by a grant from the Dutch Ministry of Health, Welfare and Sport through the Centre for Infectious Disease Control of the National Institute for Public Health and the Environment.
CLINICAL CENTRES
* denotes site coordinating physician
Amsterdam UMC, AMC site, Amsterdam: HIV treating physicians: M. van der Valk*, S.E. Geerlings, A. Goorhuis, J.W. Hovius, B. Lempkes, F.J.B. Nellen, T. van der Poll, J.M. Prins, P. Reiss, M. van Vugt, W.J. Wiersinga, F.W.M.N. Wit. HIV nurse consultants: M. van Duinen, J. van Eden, A. Hazenberg, A.M.H. van Hes, F.J.J. Pijnappel, S.Y. Smalhout, A.M. Weijsenfeld. HIV clinical virologists/chemists: S. Jurriaans, N.K.T. Back, H.L. Zaaijer, B. Berkhout, M.T.E. Cornelissen, C.J. Schinkel, K.C. Wolthers. Amsterdam UMC, VUmc site, Amsterdam: HIV treating physicians: E.J.G. Peters*, M.A. van Agtmael, M. Bomers, K.C.E. Sigaloff. HIV nurse consultants: M. Heitmuller, L.M. Laan. HIV clinical virologists/chemists: C.W. Ang, R. van Houdt, M. Jonges, J. van Prehn. Admiraal De Ruyter Ziekenhuis, Goes: HIV treating physicians: M. van den Berge, A. Stegeman. HIV nurse consultants: S. Baas, L. Hage de Looff. HIV clinical virologists/chemists: B. Wintermans, J. Veenemans. Catharina Ziekenhuis, Eindhoven: HIV treating physicians: M.J.H. Pronk*, H.S.M. Ammerlaan. HIV nurse consultants: E.S. de Munnik. HIV clinical virologists/chemists: A.R. Jansz, J. Tjhie, M.C.A. Wegdam, B. Deiman, V. Scharnhorst. DC Klinieken Lairesse - Hiv Focus Centrum: HIV treating physicians: A. van Eeden*, M. van der Valk. HIV nurse consultants: W. Brokking, L.J.M. Elsenburg, H. Nobel. HIV clinical virologists/chemists: M. Damen. ETZ (Elisabeth-TweeSteden Ziekenhuis), Tilburg: HIV treating physicians: M.E.E. van Kasteren*, M.A.H. Berrevoets, A.E. Brouwer. HIV nurse consultants: A. Adams, B.A.F.M. de Kruijf-van de Wiel, S. Keelan-Pfaf, B. van de Ven. Data collection: B.A.F.M. de Kruijf-van de Wiel, B. van der Ven. HIV clinical virologists/chemists: A.G.M. Buiting, J.L. Murck, D. Versteeg. Erasmus MC, Rotterdam: HIV treating physicians: M.E. van der Ende*, H.I. Bax, E.C.M. van Gorp, J.L. Nouwen, B.J.A. Rijnders, C.A.M. Schurink, A. Verbon, T.E.M.S. de Vries-Sluijs, N.C. de Jong-Peltenburg. HIV nurse consultants: N. Bassant, J.E.A. van Beek, M. Vriesde, L.M. van Zonneveld. Data collection: H.J. van den Berg-Cameron, J. de Groot. HIV clinical virologists/chemists: C.A.B. Boucher, M.P.G Koopmans, J.J.A van Kampen. Flevoziekenhuis, Almere: HIV treating physicians: J. Branger*, R.A. Douma. HIV nurse consultant: C.J.H.M. Duijf-van de Ven. HagaZiekenhuis, Den Haag: HIV treating physicians: E.F. Schippers*, C. van Nieuwkoop. HIV nurse consultants: J.M. van IJperen, J. Geilings. Data collection: G. van der Hut. HIV clinical virologist/chemist: N.D. van Burgel. HMC (Haaglanden Medisch Centrum), Den Haag: HIV treating physicians: E.M.S. Leyten*, L.B.S. Gelinck, F. Mollema. HIV nurse consultants: S. Davids-Veldhuis, G.S. Wildenbeest. HIV clinical virologists/chemists: E. Heikens. Isala, Zwolle: HIV treating physicians: P.H.P. Groeneveld*, J.W. Bouwhuis, A.J.J. Lammers. HIV nurse consultants: S. Kraan, A.G.W. van Hulzen, M.S.M. Kruiper. Data collection: G.L. van der Bliek, P.C.J. Bor. HIV clinical virologists/chemists: P. Bloembergen, M.J.H.M. Wolfhagen, G.J.H.M. Ruijs. Leids Universitair Medisch Centrum, Leiden: HIV treating physicians: F.P. Kroon*, M.G.J. de Boer, H. Scheper, H. Jolink. HIV nurse consultants: W. Dorama, N. van Holten. HIV clinical virologists/chemists: E.C.J. Claas, E. Wessels. Maasstad Ziekenhuis, Rotterdam: HIV treating physicians: J.G. den Hollander*, K. Pogany, A. Roukens. HIV nurse consultants: M. Kastelijns, J.V. Smit, E. Smit, D. Struik-Kalkman, C. Tearno. Data collection: T. van Niekerk. HIV clinical virologists/chemists: O. Pontesilli. Maastricht UMC+, Maastricht: HIV treating physicians: S.H.

Lowe*, A.M.L. Oude Lashof, D. Posthouwer. HIV nurse consultants: R.P. Ackens, K. Burgers, J. Schippers. Data collection: B. Weijenberg-Maes. HIV clinical virologists/chemists: I.H.M. van Loo, T.R.A. Havenith. MC Slotervaart, Amsterdam: HIV treating physicians: S.M.E. Vrouenraets*, F.N. Lauw. HIV nurse consultants: M.C. van Broekhuizen, D.J. Vlasblom, M. Kroeze. HIV clinical virologists/chemists: P.H.M. Smits. MC Zuiderzee, Lelystad: HIV treating physicians: S. Weijer*, R. El Moussaoui. HIV nurse consultant: A.S. Bosma. Medisch Centrum Leeuwarden, Leeuwarden: HIV treating physicians: M.G.A.van Vonderen*, L.M. Kampschreur. HIV nurse consultants: S. Faber, R. Steeman-Bouma. HIV clinical virologists/chemists: J Weel. Medisch Spectrum Twente, Enschede: HIV treating physicians: G.J. Kootstra*, C.E. Delsing. HIV nurse consultants: M. van der Burg-van de Plas, H. Heins. Noordwest Ziekenhuisgroep, Alkmaar: HIV treating physicians: W. Kortmann*, G. van Twillert*, R. Renckens. HIV nurse consultant and data collection: D. Ruiter-Pronk, F.A. van Truijen-Oud. HIV clinical virologists/chemists: J.W.T. Cohen Stuart, ER. Jansen, M. Hoogewerf, W. Rozemeijer, W. A. van der Reijden, J.C. Sinnige. OLVG, Amsterdam: HIV treating physicians: K. Brinkman*, G.E.L. van den Berk, W.L. Blok, P.H.J. Frissen, K.D. Lettinga W.E.M. Schouten, J. Veenstra. HIV nurse consultants: C.J. Brouwer, G.F. Geerders, K. Hoeksema, M.J. Kleene, M. Knapen, I.B. van der Meché, E. Mulder-Seeleman, A.J.M. Toonen, S. Wijnands. HIV clinical virologists: D. Kwa. Radboudumc, Nijmegen: HIV treating physicians: R. van Crevel*, A.S.M. Dofferhoff, H.J.M. ter Hofstede, J. Hoogerwerf, M. Keuter, O. Richel. HIV nurse consultants: M. Albers, K.J.T. Grintjes-Huisman, M. de Haan, M. Marneef, R. Strik-Albers. HIV clinical virologists/chemists: J. Rahamat-Langendoen, F.F. Stelma. HIV clinical pharmacology consultant: D. Burger. Rijnstate, Arnhem: HIV treating physicians: E.H. Gisolf*, R.J. Hassing, M. Claassen. HIV nurse consultants: G. ter Beest, P.H.M. van Bentum, N. Langebeek. HIV clinical virologists/chemists: R. Tiemessen, C.M.A. Swanink. Spaarne Gasthuis, Haarlem: HIV treating physicians: S.F.L. van Lelyveld*, R. Soetekouw. HIV nurse consultants: L.M.M. van der Prijt, J. van der Swaluw. Data collection: N. Bermon. HIV clinical virologists/chemists: W.A. van der Reijden, R. Jansen, B.L. Herpers, D.Veenendaal. Medisch Centrum Jan van Goyen, Amsterdam: HIV treating physicians: D.W.M. Verhagen. HIV nurse consultants: M. van Wijk. Universitair Medisch Centrum Groningen, Groningen: HIV treating physicians: W.F.W. Bierman*, M. Bakker, J. Kleinnijenhuis, E. Kloeze, A. Middel, Y. Stienstra, C.L. Vermont, KM. Wouthuyzen-Bakker. HIV nurse consultants: A. Boonstra, H. de Groot-de Jonge, P.A. van der Meulen, D.A. de Weerd. HIV clinical virologists/chemists: H.G.M. Niesters, C.C. van Leer-Buter, M. Knoester. Universitair Medisch Centrum Utrecht, Utrecht: HIV treating physicians: A.I.M. Hoepelman*, J.E. Arends, R.E. Barth, A.H.W. Bruns, P.M. Ellerbroek, T. Mudrikova, J.J. Oosterheert, M.J.A. de Regt, E.M. Schadd, M.A.D. van Zoelen. HIV nurse consultants: K. Aarsman, B.M.G. Griffioen-van Santen, I. de Kroon, C.S.A.M. van Rooijen. Data collection: M. van Berkel, C.S.A.M. van Rooijen. HIV clinical virologists/chemists: R. Schuurman, F. Verduyn-Lunel, A.M.J. Wensing.
Coordinating center. Director: P. Reiss. Deputy director: S. Zaheri. Data analysis: A.C. Boyd, D.O. Bezemer, A.I. van Sighem, C. Smit, F.W.M.N. Wit. Data management and quality control: M. Hillebregt, A. de Jong, T. Woudstra. Data monitoring: D. Bergsma, R. Meijering, T. Rutkens. Data collection: L. de Groot, M. van den Akker, Y. Bakker, A. El Berkaoui, M. Bezemer, N. Brétin, E. Djoechro, J. Geerlinks, E. Kruijne, C. Lodewijk, E. Lucas, R. van der Meer, L. Munjishvili, F. Paling, B. Peeck, C. Ree, R. Regtop, Y. Ruijs, L. van de Sande, M. Schoorl, P. Schnörr, E. Tuijn, L. Veenenberg, S. van der Vliet, E.C. Witte. Patient registration: B. Tuk.

Boston Medical Center Cohort

PI: Sara Lodi, Boston University School of Public Health. The goal of this newly established cohort is to examine clinical and public health questions about HIV disease, hepatitis C disease and substance abuse, and their intersection in era of the opioid epidemic. The Boston Medical Center is the largest safety net hospital in New England and it functions as the primary site of care for a diverse urban population comprising groups with typical high prevalence of HCV infection, HIV infection, and substance abuse such as the homeless and low-income patients.

The database consists of electronic medical records collected in routine clinical practice at the Boston Medical Center. The cohort includes adult individuals with HIV infection, chronic hepatitis C, and/or a diagnostic code for opioid use disorder between 1/1/2005 and 1/1/2018. Data collection includes all diagnostic codes, prescriptions, procedures, laboratory test dates, and results; self-reported smoking habits, alcohol use, and substance use; risk group for HIV-positive individuals, death, and emergency room visits. The database was pooled in June 2018. The cohort includes approximately 8,000 HIV-

positive individuals and 13,000 individuals with chronic HCV infection. Approximately one third of the individuals with chronic HCV infection also have a diagnostic code for opioid use disorder. Quality control is conducted through multiple data checks and resolution is reached with the help of Boston Medical Center clinicians. The database is managed at Boston University School of Public Health. Funding: Boston University School of Public Health – Pilot Award.

Canadian HIV-Hepatitis C co-infection cohort
PI: Marina Klein, McGill University, Montreal, Canada.
The Canadian Co-infection Cohort (CCC) is a prospective observational study that actively follows people living with both HIV and hepatitis C (HCV). Established in 2003, the CCC recruits from 18 centers across Canada and is active in the provinces of British Columbia, Alberta, Saskatchewan, Ontario, Quebec, and Nova Scotia. Sites are in large urban centers and smaller cities, in hospital clinics, community care, and outreach settings, making the CCC participants a diverse real-world HIV-HCV population.
Since 2016 the primary focus of the cohort has been to monitor the scale up and impacts of direct-acting antiviral (DAA) medications for hepatitis treatment among co-infected Canadians. Prior to 2016, the cohort aimed to investigate means of slowing liver disease progression rates in HIV-HCV co-infection and evaluate the role of HIV and HCV treatment in the evolution of liver disease with a particular emphasis on evaluating access to treatment, predictors of response, and comparing treatment responders vs. non-responders.
Collected data: Visits are scheduled every 6 months (±1 month) specifically for the study or incorporated into routine medical follow-up for a minimum of 5 years. Socio-demographic, medical, behavioral, and quality of life information are collected using questionnaires. Clinical endpoints and deaths are carefully collected and reviewed. Detailed information on causes of death is collected using the Coding of Death in HIV (CoDe) system.

The Canadian Coinfection Cohort investigators (CTN222) are: Drs Lisa Barrett, QEII Health Science Centre for Clinical Research, Halifax, NS; Jeff Cohen, Windsor Regional Hospital Metropolitan Campus, Windsor, ON; Brian Conway, Vancouver Infectious Diseases Research and Care Centre, Vancouver, BC; Curtis Cooper, Ottawa Hospital Research Institute, Ottawa, ON; Pierre Côté, Clinique du Quartier Latin, Montréal, QC; Joseph Cox, MUHC IDTC Montréal General Hospital, Montréal, QC; John Gill, Southern Alberta HIV Clinic, Calgary, AB; Shariq Haider, McMaster University, Hamilton, ON; Mark Hull, BC Centre for Excellence in HIV/AIDS, Vancouver, BC; Marina Klein, McGill University Health Centre, Division of Infectious Diseases and Chronic Viral Illness Service, Montreal, QC; Julio Montaner, St. Paul's Hospital, Vancouver, BC; Erica Moodie, McGill University, Montreal, QC; Neora Pick, Oak Tree Clinic, Children's and Women's Health Centre of British Columbia, University of British Columbia, Vancouver; Valerie Martel-Laferiere, Centre Hospitalier de l'Universite de Montreal, Montreal, QC; Roger Sandre, HAVEN Program, Sudbury, ON; Steve Sanche, SHARE University of Saskatchewan, Saskatoon, SK; Marie-Louise Vachon, Centre Hospitalier Universitaire de Québec, Québec, QC; Sharon Walmsley, University Health Network, Toronto, ON; Alex Wong, Regina Qu'Appelle Health Region, Regina General Hospital, Regina, SK.; and David Wong, University Health Network, Toronto, ON.

Financial support. This work was supported by Fonds de recherche du Québec—Santé (FRQ-S); Réseau SIDA/maladies infectieuses, the Canadian Institute for Health Research (CIHR) (FDN-143270) and the CIHR
Canadian HIV Trials Network (CTN222). M. B. K. is supported by a Tier I Canada Research Chair.

CoRIS/CoRIS-MD
CoRIS is funded by the SPANISH AIDS Research Network (RIS) RD16/0025/0001 project as part of the Plan Nacional R + D + I and cofinanced by ISCIII- Subdirección General de Evaluación y el Fondo Europeo de Desarrollo Regional (FEDER). Steering committee: S Moreno, J del Amo, D Dalmau, ML Navarro, MI González, JL Blanco, F Garcia, R Rubio, JA Iribarren, F Gutiérrez, F Vidal, J Berenguer, J González. Field work, data management, and statistical analyses: P Sobrino, I Jarrín, B Alejos, V Hernando, D Alvarez, C Moreno. Participating centres: Hospital General Universitario de Alicante,

Alicante (J Portilla, E Merino, S Reus, V Boix, L Giner, C Gadea, I Portilla, M Pampliega, M Díez, JC Rodríguez, J Sánchez-Payá) ; Hospital Universitari de Bellvitge, Badalona (D Podzamczer, E Ferrer, A Imaz, E Van Den Eyncle, S Di Yacovo, M Sumoy); Hospital Universitario de Canarias, Santa Cruz de Tenerife (JL Gómez, J Hernández, MR Alemán, MM Alonso, MI Hernández, F Díaz-Flores, D García, R Pelazas) ; Hospital Universitario Central de Asturias, Oviedo (V Asensi, E Valle, JA Cartón); Hospital Clínico San Carlos, Madrid (V Estrada, MJ Téllez, J Vergas, E Pérez-Cecila); Hospital Doce de Octubre, Madrid (R Rubio, F Pulido, O Bisbal, M Matarranz, M Lagarde, R Rubio-Martín, A Hernando, L Bermejo, L Dominguez); Hospital Universitario Donostia, San Sebastián (JA Iribarren, J Arrizabalaga, MJ Aramburu, X Camino, F Rodríguez-Arrondo, MÁ von Wichmann, L Pascual, MÁ Goenaga, MJ Bustinduy, H Azkune, M Ibarguren, M Aguado, M Umerez); Hospital General Universitario de Elche, Elche (F Gutiérrez, M Masiá, C López, S Padilla, A Navarro, F Montolio, C Robledano, JG Colomé, A Adsuar, R Pascual, F Carlos, M Martinez, J Llenas, M Fernández, E García); Hospital Germans Trías i Pujol, Badalona (R Muga, J Tor, A Sanvisens); Hospital General Universitario Gregorio Marañón, Madrid (J Berenguer, JC López Bernaldo de Quirós, P Miralles, I Gutiérrez, M Ramírez, B Padilla, P Gijón, A Carrero, T Aldamiz-Echevarría, F Tejerina, FJ Parras, P Balsalobre, C Diez; Hospital Universitari de Tarragona Joan XXIII, IISPV, Universitat Rovira i Virgili, Tarragona (F Vidal, J Peraire, C Viladés, S Veloso, M Vargas, M López-Dupla, M Olona, A Aguilar, JJ Sirvent, V Alba, O Calavia; Hospital Universitario La Fe, Valencia (M Montero, J Lacruz, M Blanes, E Calabuig, S Cuellar, J López, M Salavert) ; Hospital Universitario La Paz/IdiPaz, Madrid (J González, I Bernardino, JR Arribas, ML Montes, JM Peña, B Arribas, JM Castro, FJ Zamora, I Pérez, M Estébanez, S García, M Díaz, NS Alcáriz, J Mingorance, D Montero, A González, MI de José); Hospital de la Princesa, Madrid (I de los Santos, J Sanz, A Salas, C Sarriá, A Gómez-Berrocal, L Garcia-Fraile; Hospital San Pedro-CIBIR, Logroño (JA Oteo, JR Blanco, V Ibarra, L Metola, M Sanz, L Pérez-Martínez) ; Hospital Universitario Miguel Servet, Zaragoza (A Pascual, C Ramos, P Arazo, D Gil); Hospital Universitari Mutua de Terrassa, Terrassa (D Dalmau, A Jaén, M Cairó, D Irigoyen, Q Jordano, M Xercavins, J Martinez-Lacasa, P Velli, R Font, M Sanmartí, L Ibáñez; Complejo Hospitalario de Navarra, Pamplona (M Rivero, MI Casado, JA Díaz, J Uriz, J Repáraz, C Irigoyen, MJ Arraiza); Hospital Parc Taulí, Sabadell (F Segura, MJ Amengual, G Navarro, M Sala, M Cervantes, V Pineda, V Segura, M Navarro, E Antón, MM Nogueras); Hospital Ramón y Cajal, Madrid (S Moreno, JL Casado, F Dronda, A Moreno, MJ Pérez Elías, D López, C Gutiérrez, N Madrid, A Lamas, P Martí, A de Diaz, S Serrrano, L Donat); Hospital Reina Sofía, Murcia (A Cano, E Bernal, Á Muñoz); Hospital San Cecilio, Granada (F García, J Hernández, A Peña, L Muñoz, J Parra, M Alvarez, N Chueca, V Guillot, D Vinuesa, JA Fernández); Centro Sanitario Sandoval, Madrid (J Del Romero, C Rodríguez, T Puerta, JC Carrió, M Vera, J Ballesteros); Hospital de la Santa Creu i Sant Pau, Barcelona (P Domingo, MA Sambeat, K Lamarca, G Mateo, M Gutiérrez, I Fernández); Hospital Universitario Santiago de Compostela, Santiago de Compostela (A Antela, E Losada); Hospital Son Espases, Palma de Mallorca (M Riera, M Peñaranda, M Leyes, MA Ribas, AA Campins, C Vidal, L Gil, F Fanjul, C Marinescu); Hospital Universitari Vall d´Hebron, Barcelona (E Ribera); Hospital Virgen de la Victoria, Málaga (J Santos, M Márquez, I Viciana, R Palacios, I Pérez, CM González); Hospital Universitario Virgen del Rocío, Sevilla (P Viciana, M Leal, LF López-Cortés, N Espinosa); Hospital Universitario de Basurto, Bilbao (J Muñoz, M Zuriñe Zubero, J Mirena, S Ibarra, O Ferrero, J López de Munain, MM Cámara, I López, M de la Peña); Hospital Universitario Infanta Sofía, San Sebastián de los Reyes (I Suárez-García, E Malmierca); Hospital Universitario Costa del Sol, Marbella (J Olalla, A del Arco, J de la Torre, JL Prada, Z Caracuel); Hospital del Poniente, El Ejido (AM Lopez-Lirola, AB Lozano, E Fernández, I Pérez, JM Fernández); Hospital Universitario Santa Lucia, Cartagena (OJ Martínez, FJ Vera, L Martínez, J García, B Alcaraz, A Jimeno); INIBIC-Complejo Hospitalario Universitario de A Coruña, A Coruña (E Poveda, B Pernas, A Mena, M Grandal, A Castro, JD Pedreira); Hospital Clínico Universitario Virgen de la Arrixaca, Murcia (C Galera, H Albendin, A Iborra, A Moreno, MA Campillo, A Vidal); Hospital Marina Baixa, Villajoyosa (C Amador, F Pasquau, J Ena, C Benito, V Fenoll); Complejo Hospitalario de Jaén, Jaén (MO Mohamed-Balghata, MA Gómez); Hospital San Agustín de Avilés, Avilés (MA de Zarraga, ME Rivas); Fundación Jiménez Diaz,Madrid (M Górgolas).

ICONA

PI: Antonella D'Arminio Monforte - Institute of Infectious and Tropical Diseases, Department of Health Sciences; University of Milan, Italy

The Icona Foundation Study cohort is an observational multicentre cohort that enrolls HIV-infected individuals who are antiretroviral-naïve at the time of enrollment. Patients are voluntary enrolled by physicians at the different centers in Italy participating in ICONA Study after signing an informed consent. This cohort was set up in January 1997 and currently includes data on patients enrolled at 51 infectious disease units in Italy. The ICONA database includes all events over the follow-up (i.e., all laboratory measurements, clinical events, medication and treatment changes, hospitalizations, and death); in their absence, a follow-up visit is scheduled at least every 6 months.

Southern Alberta Clinic Cohort

PI: Dr. John Gill, Southern Alberta HIV Clinic, Canada. The Southern Alberta Clinic Cohort is a geographically defined clinical cohort of all HIV infected patients receiving their HIV care in S. Alberta. The clinic is the primary site of care for 93% of HIV positive patients with 3 secondary sites serving specific populations (7%). The prospective cohort started in 1989 has ongoing rolling recruitment (retroactive data entry 1984-1989). Total number in cohort dataset was >5050 on Dec 31 2021: Female 23%, Risk groups: MSM (48%); IDUs (17%); Heterosexuals including migrants (31%); perinatal Transmission: 0.27% other or unknown (2%). Current active roster 2025. Our database/EMR collects and retains very extensive demographic, therapeutic, laboratory (HIV and non HIV), clinical , hospitalization, social, public health, and cost data on every encounter of our patients within the Province. The clinic has over 400 peer reviewed publications. Current research includes mortality and causes of death, toxicity and adverse event studies, economic and outcome analysis, HIV Epidemiology, resistance in therapy naive patients, acute seroconversion illness , HIV phylogenetics , adherence, intimate partner violence and HIV, churn effects, HIV and housing, and treatment outcomes.

SWISS HIV COHORT STUDY

The Swiss HIV Cohort study is funded by the Swiss National Science Foundation grant # 148522, by the SHCS research foundation. The SHCS drug resistance database is supported by the Yvonne Jacob Foundation and by the SNF grant # 179571.

MEMBERS. Anagnostopoulos A, Battegay M, Bernasconi E, Böni J, Braun DL, Bucher HC, Calmy A, Cavassini M, Ciuffi A, Dollenmaier G, Egger M, Elzi L, Fehr J, Fellay J, Furrer H (Chairman of the Clinical and Laboratory Committee), Fux CA, Günthard HF (President of the SHCS), Haerry D (deputy of "Positive Council"), Hasse B, Hirsch HH, Hoffmann M, Hösli I, Huber M, Kahlert C, Kaiser L, Keiser O, Klimkait T, Kouyos RD, Kovari H, Ledergerber B, Martinetti G, Martinez de Tejada B, Marzolini C, Metzner KJ, Müller N, Nicca D, Paioni P, Pantaleo G, Perreau M, Rauch A (Chairman of the Scientific Board), Rudin C (Chairman of the Mother & Child Substudy), Scherrer AU (Head of Data Centre), Schmid P, Speck R, Stöckle M, Tarr P, Trkola A, Vernazza P, Wandeler G, Weber R, Yerly S.

Veterans Aging Cohort Study-HIV (VACS-HIV)

PI: Amy C. Justice, VA Connecticut Healthcare System, Yale University School of Medicine
Based on the US Veterans Administration (VA) Healthcare System Electronic Medical Record, VACS-HIV is an ongoing observational cohort study of approximately 60,000 people with HIV (PWH) and 120,000 age/race/sex/site matched uninfected participants with over 20 years of follow up. The study's aim is to understand the role of comorbid medical and psychiatric disease and substance use in determining clinical outcomes in HIV infection. It is funded primarily by the National Institute on Alcoholism and Alcohol Abuse, National Institutes of Health, with in-kind VA support and has a special focus on the role of alcohol use and misuse.
Website: vacohort.org

Parametric g-formula

The g-formula is a generalization of standardization to the time-varying setting. When the measured variables are sufficient to adjust for confounding and selection bias [1, 2], the g-formula under a specified intervention identifies the outcome distribution in the study population had that intervention been, possibly contrary to fact, implemented in all individuals in the population. The parametric g-formula is an approach to estimate the components of the g-formula using parametric models [3-5]. For this study, the outcome was sustained virological response (SVR) and the intervention was receiving an HCV-RNA test between 10 and 24 months after end of treatment (SVR assessment window) under a prespecified frequency of HCV-RNA testing in the SVR assessment window. The parametric g-formula algorithm has two steps. In the first step, we fit parametric regression models to estimate the distribution of outcome and time-varying covariates in the SVR assessment window conditional on prior history. Second, we standardize the probability of the outcome to the conditional distributions, both estimated in step 1, under the intervention values.

For the first step, we fit separate logistic regression models for time-varying indicators at time t (HCV-RNA test, ALT test, AST test, and platelet count test) and linear regression models for continuous variables on the natural logarithm scale (AST, ALT, and platelet count). All these models included as covariates the most recent value of the time-varying covariates (time since end DAA treatment, time since last HCV-RNA test, AST, ALT, platelet count) and baseline covariates age (<35,35-50,>50 years), sex, mode of HIV acquisition, cohort, calendar period, CD4 cell count category (<350, 350-500,>500 cells/mm3), HIV virological suppression (HIV-RNA ≤50, >50 copies/mL), history of antiretroviral treatment for HIV, history of AIDS, hepatitis B virus co-infection (presence of either hepatitis B surface antigen or detectable hepatitis B virus DNA), prior HCV treatment with interferon, HCV genotype, and fibrosis stage categorized as no significant fibrosis (FIB-4<1.45), significant fibrosis (FIB-4≥1.45 and FIB-4≤3.25), and cirrhosis (FIB-4>3.25).

For the second step, because the standardization involves a complex sum, we approximate it using Monte Carlo simulation. We then estimated the population SVR proportion as the average of the subject-specific probability of achieving SVR by the end of the SVR assessment window.

Finally, we used a nonparametric bootstrap procedure based on 500 samples to obtain percentile-based 95% confidence intervals.

References

[1] J. M. Robins, "A new approach to causal inference in mortality studies with a sustained exposure period: application to the healthy worker survivor effect.," *Mathematical Modelling,* vol. 7, no. 9-12, pp. 1393-1512, 1986.

[2] M. A. Hernán and J. M. Robins, *Causal Inference: What if*, Forthcoming ed. Boca Raton: Chapman & Hall/CRC, 2020.

[3] S. Lodi, A. Phillips, R. Logan, A. Olson, D. Costagliola, S. Abgrall *et al.*, "Comparative effectiveness of strategies for antiretroviral treatment initiation in HIV-positive individuals in high-income countries: an observational cohort study of immediate universal treatment versus CD4-based initiation," *Lancet HIV,* vol. 2, no. 8, pp. e335–e343, 2015. [Online]. Available: http://dx.doi.org/10.1016/S2352-3018(15)00108-3.

[4] J. G. Young, L. E. Cain, J. M. Robins, E. J. O'Reilly, and M. A. Hernan, "Comparative effectiveness of dynamic treatment regimes: an application of the parametric g-formula," (in Eng), *Stat Biosci,* vol. 3, no. 1, pp. 119-143, Sep 1 2011, doi: 10.1007/s12561-011-9040-7.

[5] S. L. Taubman, J. M. Robins, M. A. Mittleman, and M. A. Hernan, "Intervening on risk factors for coronary heart disease: an application of the parametric g-formula," *Int J Epidemiol,* vol. 38, no. 6, pp. 1599-611, Dec 2009, doi: 10.1093/ije/dyp192.

Appendix table 1

Appendix Table 1. Baseline characteristics of 4527 patients with hepatitis C and HIV co-infection who initiated direct acting antiviral (DAA) treatment by cohort study. HepCAUSAL 2014-202

Cohort	Eligible	SVR assessed	Male	Age<50	FIB-4>3.25	Year 2014-2016	At least one HCV-RNA test during DAA treatment	CD4<=350
AMACS	11	2 (18%)	11 (100%)	7 (64%)	1 (9%)	6 (55%)	0	1 (9%)
AQUITAINE	260	248 (85%)	192 (74%)	101 (39%)	41 (216%)	208 (80%)	244 (94%)	34 (13%)
ATHENA	333	261 (78%)	310 (93%)	231 (69%)	27 (8%)	312 (94%)	233 (70%)	33 (10%)
BMC	150	109 (73%)	111 (74%)	34 (23%)	40 (27%)	124 (83%)	119 (79%)	34 (23%)
CCC	499	463 (93%)	359 (72%)	240 (48%)	102 (20%)	281 (56%)	446 (89%)	142 (28%)
CORIS	181	147 (81%)	148 (82%)	113 (62%)	31 (17%)	173 (96%)	145 (80%)	37 (20%)
ICONA	370	299 (81%)	287 (76%)	155 (42%)	70 (19%)	132 (36%)	288 (78%)	56 (15%)
SHCS	381	249 (65%)	269 (71%)	139 (36%)	102 (27%)	296 (78%)	273 (72%)	66 (17%)
VACS	2342	2107 (90%)	2309 (98%)	162 (7%)	62 (28%)	2311 (98%)	2213 (95%)	522 (22%)
